# Reduction of EpCAM-Positive Cells from a Cell Salvage Product Is Achieved by Leucocyte Depletion Filters Alone

**DOI:** 10.3390/jcm12124088

**Published:** 2023-06-16

**Authors:** Lucia Merolle, Davide Schiroli, Daniela Farioli, Agnese Razzoli, Gaia Gavioli, Mauro Iori, Vando Piccagli, Daniele Lambertini, Maria Chiara Bassi, Roberto Baricchi, Chiara Marraccini

**Affiliations:** 1 Transfusion Medicine Unit, Azienda USL-IRCCS di Reggio Emilia, 42123 Reggio Emilia, Italy; 2 Clinical and Experimental Medicine PhD Program, University of Modena and Reggio Emilia, 41125 Modena, Italy; 3 Medical Physics Unit, Azienda USL—IRCCS di Reggio Emilia, 42123 Reggio Emilia, Italy; 4 Medical Library, Azienda USL—IRCCS di Reggio Emilia AUSL-IRCCS di Reggio Emilia, 42123 Reggio Emilia, Italy

**Keywords:** cell salvage, cancer surgery, leucocyte depletion filters, irradiation, cancer cells

## Abstract

Intraoperative cell salvage reduces the need for allogeneic blood transfusion in complex cancer surgery, but concerns about the possibility of it re-infusing cancer cells have hindered its application in oncology. We monitored the presence of cancer cells on patient-salvaged blood by means of flow cytometry; next, we simulated cell salvage, followed by leucodepletion and irradiation on blood contaminated with a known amount of EpCAM-expressing cancer cells, assessing also residual cancer cell proliferation as well as the quality of salvaged red blood cell concentrates (RBCs). We observed a significant reduction of EpCAM-positive cells in both cancer patients and contaminated blood, which was comparable to the negative control after leucodepletion. The washing, leucodepletion and leucodepletion plus irradiation steps of cell salvage were shown to preserve the quality of RBCs in terms of haemolysis, membrane integrity and osmotic resistance. Finally, cancer cells isolated from salvaged blood lose their ability to proliferate. Our results confirm that cell salvage does not concentrate proliferating cancer cells, and that leucodepletion allows for the reduction of residual nucleated cells, making irradiation unnecessary. Our study gathers pieces of evidence on the feasibility of this procedure in complex cancer surgery. Nevertheless, it highlights the necessity of finding a definitive consensus through prospective trials.

## 1. Introduction

Allogeneic blood transfusion is potentially life-saving for bleeding patients, but it is not totally risk-free [1]. Intraoperative cell salvage, also known as autotransfusion, is a strategy designed to reduce the need for allogeneic transfusions. This procedure involves the collection of patient blood from the surgical field, which is usually centrifugally washed to remove non-cellular matter and re-infused back into the same person. Red blood cells washing devices provide high-quality blood components with negligible side effects [2]. Cell salvage is widely used in major orthopaedics and vascular surgery to reduce or prevent allogeneic transfusion and perfectly fits in the scenario of Patient Blood Management (PBM) programs [3]. However, cell salvage is often not used in cancer surgery due to concerns dating back to the 1970s, when a case report on a lung cancer patient described the presence of residual cancer cells in salvaged blood [4]. Since then, many case reports, editorials and in vitro studies have focused on warnings about using this technique on cancer patients, reporting the results of several experiments that evidenced the presence of residual cancer cells in salvaged blood (and sometimes even more concentrated than those present in patient peripheral blood) [5,6]. The main concern, therefore, regarded the possibility of re-infusing to the patient cancer cells that might have eventually spilled out from the tumor site, thereby contributing to the risk of metastasis [7,8,9]. Despite the concerns, growing literature on both in vitro and clinical studies support the use of cell salvage in cancer surgery [10,11,12,13,14].

Based on the recommendations of national and international scientific societies, cell salvage can be applied to cancer surgery, taking into account local protocols, patient characteristics and medical team expertise. Salvaged blood could theoretically be re-infused as it is, but guidelines worldwide usually encourage its use in combination with leucodepletion filters [15], which, according to the most precautionary approaches [16], should also be followed by irradiation before reinfusion. Compared to the less efficient leucoreduction filters [17,18], leucocyte depletion filters have been successfully used to deplete cancer cells from salvaged blood [19,20,21] and are highly compatible with an intra-operative setting. On the other hand, irradiation implies some organizational criticisms that hinder the intraoperative recovery and washing of patient blood.

Given these premises, we tested the ability of a cell salvage system to reduce cancer cells that are eventually released to the surgical field, to the suction reservoir and into the autologous blood product during surgery. First, we assessed the efficiency of cancer cell reduction from the autologous blood product obtained from cancer patients undergoing partial haepatectomy upon cell salvage followed by leucodepletion (without subsequent reinfusion). Next, we simulated cell salvage followed by leucodepletion and irradiation of whole blood units from healthy donors, which were inoculated with a known amount of cancer cells expressing the epithelial cell adhesion molecule (EpCAM) (HCT116 and CaCo-2 cell lines). We monitored cancer cell distribution step-by-step during the whole procedure, also assessing the proliferation capacity and overall quality of salvaged red blood cells.

The data collected were discussed in light of the recent scientific literature on cell salvage in cancer, with a specific focus on filtered and/or irradiated salvaged blood, in order to understand the real usefulness of these steps on cancer patient salvaged blood. 

## 2. Materials and Methods

### 2.1. Study Population

This study was conducted at the Azienda USL-IRCCS di Reggio Emilia (Italy). Blood was collected from cancer patients to perform cell salvage after partial hepatectomy, without subsequent reinfusion, after the approval by the local Ethics Committee in December 2015 (protocol number 2015/29613). In total, 12 cancer patients undergoing partial hepatectomy for primary hepatocarcinoma (HCC, *n* = 8) or colorectal cancer liver metastasis (*n* = 4) were recruited between July and December 2016. Salvaged blood was not re-infused and was used for research only. Details on patients’ demographic and surgery characteristics are summarized in Table 1. The collection of whole blood from 20 healthy donors for the in vitro cell salvage study was approved by the local Ethical Committee in July 2017 (protocol number 2017/0066269). All volunteer donors and patients recruited provided informed consent according to the Declaration of Helsinki.

### 2.2. Cell Salvage on Cancer Patients

**Figure 1:**
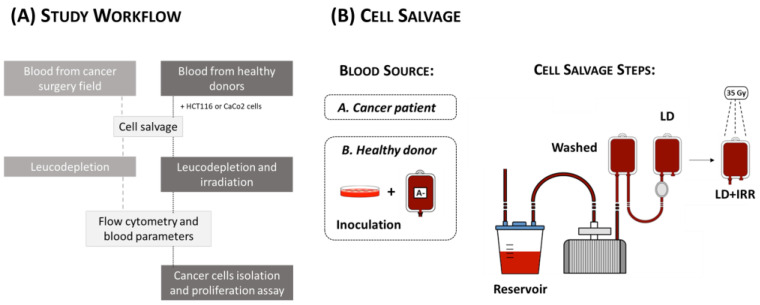
(**A**) Workflow of the study; (**B**) Scheme of the ex vivo and in vitro cell salvage steps from blood recovered from the surgical field of cancer patients (A. Cancer patient), and from healthy donors (B. Healthy donor).

Partial hepatectomy was chosen as the surgical model for cell salvage simulation since, within our institution, it is among the cancer surgeries with the highest transfusion risk, with an anticipated blood loss of 1000 mL. Patient blood samples were collected between July and December 2016 to simulate cell salvage without subsequent reinfusion. For the 12 cancer patients recruited, blood that had spilled out from the operative field was collected with a suction line connected to a continuous autotransfusion system (C.A.T.S., Fresenius HemoCare Italia S.r.l, Mirandola, Italy). The “High Quality program” was set for performing the cell salvage according to manufacturer’s instructions. Two independent blood aliquots (5 mL each in EDTA-containing vacuum tubes) were collected for each of the following time points (Figure 1B): device reservoir (Reservoir); blood unit after centrifugal washing (Washed); blood unit after leucodepletion (BIO-R flex Fresenius HemoCare Italia S.r.l.) (Leucodepleted, LD); blood unit after irradiation at 35Gy (Raycell X-ray Blood Irradiator, MDS Nordion, Ottawa, ON, Canada) (Leucodepleted + Irradiated, LD + IRR). All samples were analyzed immediately after collection. 

### 2.3. Cell Culture

The HCT116 colon cancer cell line was cultured in an IMDM GlutaMAX medium (GIBCO, ThermoFisher Scientific, Monza, Italy), while the CaCo-2 colon cancer cell line was cultured in DMEM High glucose (Euroclone SpA, Milan, Italy) supplemented with 1% L-glutamine, 10% FCS, 100 U/mL penicillin and 100 μg/mL streptomycin (GIBCO, ThermoFisher Scientific, Monza, Italy). Cultures were maintained in a humidified atmosphere with 5% CO_2_ at 37 °C. Prior to inoculation in whole blood units, cells were trypsinized, resuspended in complete medium and a small aliquot was mixed with Trypan blue solution (0.14% in HBSS). The method for distinguishing the viable from dead cells is based on the principle that live (viable) cells do not take up certain dyes, whereas dead (non-viable) cells do. Colored and dye-negative cells were counted on an automated cell counter (Countess, Invitrogen).

### 2.4. Cell Salvage In Vitro Simulation

Whole blood units from 20 healthy donors were collected in CPD solution (Citrate, Phosphate and Dextrose). A blood count was performed on 1 mL aliquots (negative control) using a Sysmex XS-1000i Hematology Analyzer (Dasit S.p.A., Milan, Italy) before cancer cell inoculation. HCT116 and CaCo-2 cells were inoculated, at known concentration, into each blood unit to match a high patient amount of circulating tumor cells [10]. Inoculated blood samples were sucked and collected within the reservoir of the C.A.T.S. automated system to simulate the suction occurring at the surgical field. Blood aliquots in EDTA-containing vacuum tubes were collected as follows (Figure 1B): inoculated whole blood before suction (Inoculation—10 mL); blood unit after cell salvage washing (Washed—10 mL); blood unit after leucodepletion (LD—10 mL); blood unit after irradiation at 35Gy (LD + IRR—10 mL). Collected samples were immediately assessed for further analyses.

### 2.5. Flow Cytometric Assessment of Circulating Tumor Cells (CTCs)

The cancer cell count was assessed by evaluating the EpCAM-associated fluorescence signal, as previously reported [21]. Staining was carried out on 100 µL aliquots with the antibodies 5 μL anti-EpCAM-PE (CD326, clone HEA-125, Miltenyi Biotec, Bologna, Italy) and 5 μL anti-CD45-FITC-conjugated antibody (Beckman Coulter) for 10 min in the dark at room temperature in order to exclude white blood cells. Before acquisition, each sample was treated with 2 mL of lysing solution (Stem-Kit Reagents, Beckman Coulter, Milan, Italy) to avoid erythrocytes interference. To perform an absolute count of EpCAM-positive (EpCAM^+^) cells, 100 µL of calibration beads (LeukoSure Fluorospherers, Beckman Coulter, Milan, Italy) were added immediately before acquisition (FC 500 flow cytometer, Beckman Coulter, Milan, Italy). Negative control samples were treated as described, without adding monoclonal fluorescent antibodies.

### 2.6. Cancer Cell Isolation and Proliferation Assay

CaCo-2 cells were isolated from the inoculation, washed, leucodepleted and irradiated as steps of the cell salvage simulation with the Ficoll Paque^®^ PLUS density gradient centrifugation. For each step, 10 mL samples were diluted 1:1 with sterile PBS; next, the blood/PBS mixture was carefully deposited with a serological pipet over 10 mL Ficoll Paque^®^ PLUS in 50 mL conical tubes. Samples were centrifuged at 400× *g* for 30 min at room temperature, without a break. Cancer cell-containing discs of mononuclear cells were collected, and EpCAM^+^ cells were counted by means of flow cytometry on a small aliquot as described earlier. EpCAM^+^ isolated cells were seeded at 20,000 cells/cm^2^ in a complete medium on 24-well plates. After a 24 h seeding, cells were washed twice in PBS and a fresh complete medium was added. At day 7 from culture, cells were trypsinized, resuspended in 100 μL PBS + 100 μL calibration beads and then counted by means of flow cytometry.

### 2.7. Blood Count, Haemolysis and Erythrocyte Osmotic Resistance

The red blood cell (RBC) count, haemoglobin (Hb, g/dL), haematocrit (HCT, %), platelets (PLTs) and white blood cell (WBC) count were measured on 1 mL aliquots from patient and donor whole blood and on salvaged samples. Analyses were carried out using a CELL-DYN Ruby Hematology Analyzer (Abbott Laboratories). Haemolysis was estimated following the Harboe direct spectrophotometric method, as already published [22]. Briefly, free haemoglobin (HbO_2_) absorbance was measured at 415 nm (ε = 512 mM^−1^ cm^−1^); the percentage of haemolysis was derived using the free Hb concentration in the supernatants, the total Hb and the HCT. Erythrocyte fragility was determined by means of the osmotic resistance test in duplicates as follows: 5 µL of blood sample was mixed with 95 µL of NaCl solution at decreasing osmolarity (from 300 mOsm—corresponding to 0.9% NaCl—to 0 mOsm—corresponding to pure water); then, samples were centrifuged at 2500× *g* for 1 min. Hb absorbance in the supernatants was measured at 560 nm in a 96-well plate (GloMax plate reader, Promega Corp., Milan, Italy). Results were plotted and fitted using a sigmoidal function as previously described [23].

### 2.8. Data Management and Statistical Analysis

Data are expressed as mean ± standard deviation (SD). A one-way ANOVA with a multi-comparison test was carried out for statistical comparisons. Differences with a *p* < 0.05 were considered statistically significant. Data analyses were developed on GraphPad Prism 7.0 (GraphPad Software Inc., Boston, MA, USA) and Excel 2010 (Microsoft).

## 3. Results

### 3.1. Cell Salvage in Cancer Patients

We collected and analyzed blood samples from 12 cancer patients undergoing partial hepatectomy, simulating cell salvage followed by leucodepletion. Of the 12 patients recruited, 8 were primarily diagnosed with hepatocarcinoma, while 4 were operated on for hepatic metastases of colorectal cancer (Table 1). For each patient, we assessed samples from the following steps: blood recovered from the surgical field and collected within the autotransfusion system reservoir (also indicated as Reservoir); red blood cells collected after cell salvage procedure (Washed); red blood cells collected after further leucodepletion (LD). Mean blood volume recovery from the surgical field was 1021 mL, which allowed the recovery of around 115 mL RBCs after washing (Table 1).

**Figure 2:**
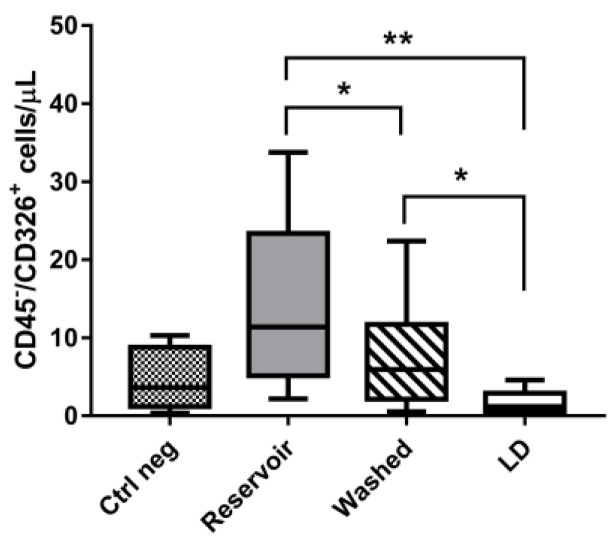
Flow cytometric count of CD45^−^/CD326^+^ (EpCAM^+^) cells (CD326^+^) in cancer patients’ blood samples recovered after the main steps of cell salvage simulation. Gating on CD45^−^ cells was performed in order to exclude WBCs. Data are expressed as mean ± SD. Significant differences among samples were assessed by one-way ANOVA; * *p* < 0.05, ** *p* < 0.01.

Flow cytometric analysis revealed that CD45^−^/EpCAM^+^ cells were present in all patient blood samples recovered from the operative filed (Reservoir), although the concentration was extremely variable (13.6 ± 10.2 mean cells/μL). For one of the patients recruited in particular, we found 41.3 ± 12.1 CD45^−^/EpCAM^+^ cells in the Reservoir, while for two other subjects the CD45^−^/EpCAM^+^ cells were less than 1 per μL (Figure 2 and Table 2). As shown in Figure 2, the simple centrifugal washing was already effective in eliminating most of the circulating EpCAM^+^ cells (7.5 ± 6.7 mean cells/μL), while leucodepletion guaranteed significant reduction in all the samples (1.7 ± 1.5 mean cells/μL after LD). Negative control samples (i.e., without anti-EpCAM-PE monoclonal antibody staining) showed a background fluorescence in the PE channel (4.5 ± 3.8 mean cells/μL) possibly attributable to the autofluorescence of the recovered blood; the non-specific bond of the antibody and the presence of solvents or disinfectants in the surgical field could have contributed to influencing flow cytometric acquisition. Nevertheless, PE fluorescence was always decreased after leucodepletion.

### 3.2. Cell Salvage In Vitro Simulation

**Figure 3:**
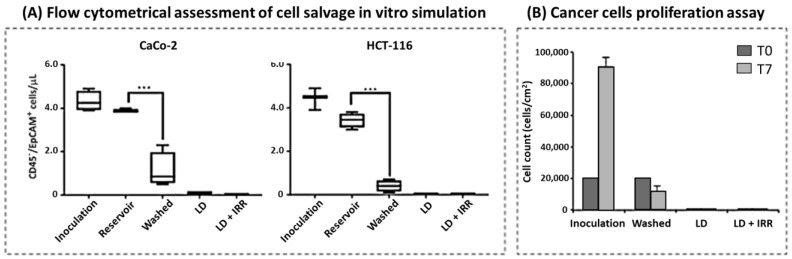
(**A**) Cancer cell counts measured by flow cytometry at each step of the cell salvage in vitro simulation. Data are expressed as mean ± SD of 10 simulations with CaCo-2 cell line (left panel) and 10 simulations with HCT116 cell line (right panel); (**B**) Cancer cell isolation and proliferation assessment by flow cytometry. T0 refers to the concentration of cells cultured immediately after isolation, while T7 refers to the concentration of cells found after 7 days of culture. Data are expressed as mean ± SD of five simulations with CaCo-2 cell line. Significant differences among samples were assessed by one-way ANOVA; *** *p* < 0.001.

Cell salvage was also simulated by taking advantage of EpCAM-expressing cancer cell lines (CaCo-2 and HCT116 colorectal cancer cell lines), which were inoculated at a known amount (40–50 cells/μL) on whole blood collected from healthy blood donors. As detailed in the Section 2, we simulated the centrifugal washing, followed by leucodepletion and subsequent γ-irradiation at 35 Gy (as indicated by Italian recommendations) [16]. Blood samples collected from these steps were first assessed by flow cytometry to monitor residual cancer cells (Figure 3A). Our results evidenced that the centrifugal washing performed by the autotransfusion system significantly reduced the concentration of inoculated cancer cells (Washed sample; CaCo-2 = 2.7 ± 2.1 cells/μL; HCT116 = 2.0 ± 1.1 cells/μL). After leucodepletion (LD sample; CaCo-2 = 0.3 ± 0.2 cells/μL; HCT116 = 0.3 ± 0.3 cells/μL), the cell counts were in the range of the negative control; these data were confirmed after irradiation (LD + IRR sample; CaCo-2 = 0.0 ± 0.0 cells/μL; HCT116 = 0.1 ± 0.1 cells/μL) for both the cell lines considered (Figure 3A). By the time of cell salvage simulation (T0, i.e., immediately after isolation) we were never able to detect EpCAM^+^ cells in LD and LD + IRR samples, while residual cells were always evident in samples from the Inoculation and Washed steps (Figure 3A).

### 3.3. Cancer Cell Isolation and Proliferation Assay

Aliquots of blood samples collected from the steps of the in vitro simulation with CaCo-2 cells were further processed to isolate and culture residual cancer cells. CaCo-2 were selected for this assay due to the high expression levels of EpCAM, which makes them more suitable for detecting very low amounts of residual cells. After density gradient stratification, mononuclear cells were collected and EpCAM^+^ cells were counted by means of flow cytometry (Figure 3B).

To assess proliferation, we cultured separated CaCo-2 cells at a concentration of 20,000 cells/cm^2^ in 24-well plates as described in the Section 2. Since no EpCAM^+^ cell was detectable on samples from LD and LD + IRR blood, we cultured the whole monolayer collected from stratification. After 7 days, cells were collected and counted by flow cytometry: results shown in Figure 3B indicate that, while cells recovered from the inoculation step kept on proliferating, washed cells did not proliferate. From both the LD and LD + IRR samples no EpCAM^+^ cell was recovered after 7 days of culture.

### 3.4. Blood Count, Haemolysis and Erythrocyte Osmotic Resistance

**Figure 4:**
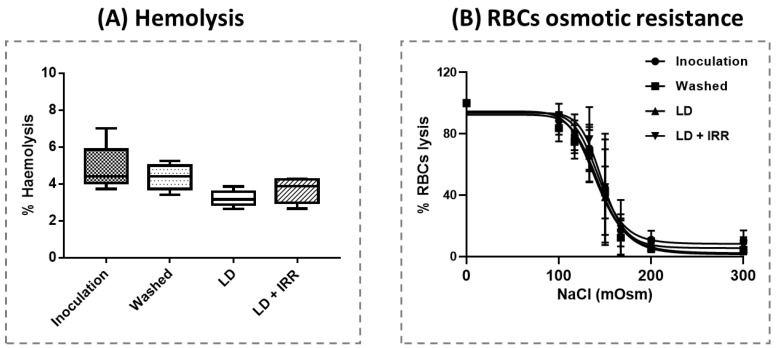
(**A**) Percentage of hemolysis of salvaged blood samples. Each value was collected in triplicate for *n* = 6 samples and expressed as mean ± SD; (**B**) Erythrocytes (RBCs) osmotic resistance test. Each value was collected in triplicate for *n* = 6 samples and expressed as mean ± SD. Points were fitted using a sigmoidal function.

Finally, blood samples collected from Washed, LD and LD+IRR steps of the cell salvage in vitro simulation were evaluated for the overall quality of RBCs. According to the blood count, haematocrit (HCT) and PLTs, all RBCs samples were in the range of blood bank allogeneic products. As expected, almost no WBCs were found in LD and LD + IRR-salvaged RBCs (see Supplementary Table S3 for details on blood count). Haemolysis was quite variable after the washing step due to the turbulence generated in the suction line during blood collection, although it was still in the range of normality (Figure 4A). Leucodepletion and irradiation did not significantly affect haemolysis. Despite the fact that RBCs during cell salvage undergo turbulence during suction, centrifugal washing, filtration and irradiation, erythrocyte resistance to haemolysis was comparable to pre-salvage whole blood, as evidenced by the erythrocyte osmotic resistance test shown on Figure 4B.

## 4. Discussion

Anemia is an independent risk factor for negative outcomes for cancer patients [24], and PBM programs for the whole perioperative period for oncological patients are becoming urgent [25]. In this context, any strategy aimed at preserving patients’ own blood and avoiding the risk of allogeneic transfusion [1,26] deserves particular consideration. Cell salvage has already demonstrated its potential in preserving patients’ own blood and in reducing the need of allogeneic transfusions, but its use in cancer surgery has long been limited due to physician concerns about reintroducing cancer cells that might have spilled out from the tumor site. To our knowledge, however, no study published since 1995 has contraindicated cell salvage use in cancer surgery, and the recent diffusion of leucodepletion filters has contributed to ensuring high quality products that encourage a timid use of auto-transfusion systems in cancer surgery [27].

With our study, we evidenced that washed samples showed a significant reduction of EpCAM+ cells, which was more evident after leucodepletion. Leucodepletion filters’ effectiveness in reducing residual cancer cells was already suggested by our ex vivo assay on cancer patients’ blood (Figure 2). The highly variable amount of EpCAM^+^ cells found in patient blood was expected, since even in metastatic patients circulating tumor cells can be quite rare and heterogeneous in terms of EpCAM expression (which can be gained and lost, especially during HCC cancer progression) [28]. Unfortunately, to date no unique marker has been developed to effectively quantify circulating cancer cells and, despite the fact that EpCAM is still considered a valuable tool, its limitations should be taken into account. CTC detection methods based on EpCAM^+^ cell quantification in cancer patients could miss those cells that, in preserving metastatic potential, lose this marker (or simply do not express it). Even when EpCAM^+^ cells are detected, the exact cancer cell count can be hindered by the complexity of blood recovered from patients with widespread metastases. In these patients, the heterogeneity of circulating cells might hamper the detection due to non-specific fluorescence signals, as we experienced for some patients recruited in the present study (see Table 2).

Given the known difficulties in detecting and isolating circulating cancer cells from peripheral blood, researchers have developed many techniques based on different principles (i.e., size exclusion, microfluidics-based cell sorting, antibody-linked functionalized nanomaterials and so on) [29]; however, all of them have pros and cons, and future detection of circulating cancer cells should involve the combination of multiple strategies simultaneously.

To overcome the limitations of detecting cancer cells from cancer patients’ blood, we moved towards an in vitro model, taking advantage of our already published method on in vitro cell salvage simulation [21]. Our data showed that cancer cells are efficiently reduced by leucodepletion filters (Figure 3A), and that this reduction persists in subsequently irradiated samples. It is noteworthy to underline that cancer cells found in the Washed steps lose their proliferation ability (Figure 3B). We hypothesize that the latter is mainly attributable to cell damage induced by a mechanical stress due the turbulence generated during blood suction and the subsequent centrifugal washing (Figure 3B). Intriguingly, damage to cancer cells is highly selective: indeed, red blood cells do not undergo cell membrane damage at any of the re-infusible steps of cell salvage (i.e., Washed, LD and LD + IRR), as evidenced by the evaluation of hemolysis and osmotic resistance (Figure 4). Cell salvage, therefore, preserves red blood cell quality, which shows similar characteristics as bank components even when coupled with filtration and irradiation (with the advantage of being autologous).

To our knowledge, this is the first in vitro study demonstrating the reduced proliferation ability of cancer cells after simulating cell salvage plus leucodepletion and irradiation. In 2016, Kumar and colleagues [30] tried to culture cancer cells recovered from the salvaged blood of metastatic spine tumor patients to investigate their viability before and after leucodepletion. Counting the cancer cell-containing samples, they observed that none of the washed and filtered samples generated clusters after culture, thereby suggesting the absence of cancer cell viability after auto-transfusion. Despite the intriguing results, the study had some limitations, as the detection method was not able to find cell cluster development for all the pre-salvage blood samples considered. Since flow cytometry is highly sensitive in detecting very low amounts of EpCAM-expressing cells [19], we believe that the results shown here represent a valuable proof in support of the hypothesis of a loss of viability of cancer cells, which occurs already after washing (Figure 3B). Furthermore, the use of a cell line with a well-established doubling time allowed us to be sure that the lower amount of cells found in the washed samples resulted from a loss of proliferation activity.

Besides in vitro analyses, it is a fact that almost no clinical study published since the 1990s has registered a negative effect from cell salvage on survival or disease progression. Most of the literature focuses on the effectiveness of cell salvage alone; however, following the worldwide diffusion of leucodepletion filters, several guidelines have recommended the use of leucodepletion filters [16,31], and some even encourage subsequent irradiation [1]. We performed a bibliographic search of the recent studies on cancer cell savage that included a leucodepletion and/or irradiation step, excluding those that did not assess at least one of these steps (see Supplementary Information for details on the string used and results obtained). Actually, the full-text original articles published since 2013 on cell salvage in combination with leucodepletion or with irradiation strategies are few and are mostly aimed at evidencing the safety of salvaged blood on in vitro models (Supplementary Table S1). Despite the fact that residual cancer cells are often recovered after centrifugal washing of both cancer patient and in vitro-contaminated blood, leucodepletion has always demonstrated its effectiveness in removing nucleated cells, thereby suggesting significant safety in clinical settings.

Studies on the effect of irradiation after cell salvage are actually rare [32,33]. In 2015, Gong M et al. detected the optimal dose to allow the complete inhibition of the viability and proliferation of cancer cells from irradiated salvaged blood by irradiating several cancer cell lines previously mixed with erythrocytes from healthy donors [31]. Nevertheless, they assessed irradiation alone and counted residual cancer cells by means of colony formation assays, which are known to be less sensitive than flow cytometry [34] or other, more recent microfluidic technologies [35] in assessing very low amounts of cells from blood samples.

The majority of clinical studies compared medium-to-long-term outcomes of cancer patients that received leucodepleted salvaged blood with control groups of patients that did not undergo cell salvage. All of them reported that leucodepleted salvaged blood is non-inferior to allogeneic transfusion in terms of mortality and recurrence rate, while it has been shown to reduce the need for postoperative allogeneic transfusion in a retrospective study on 176 metastatic spine surgery patients [36]. We found a few clinical studies comparing the outcomes after reinfusion of salvaged blood upon leucodepletion and/or irradiation; of these, only one was conducted examining the recurrence rate after autotransfusion of irradiated salvaged blood [37]. Weller and colleagues compared the tumor recurrence of HCC patients undergoing liver transplantation but, due to the small population size, they could not investigate the efficacy and safety of irradiation compared to washing or filtering.

Finally, of all the reviews published since 2012 on this topic (Supplementary Table S2), we found only one meta-analysis published in 2022, which investigated the safety of cell salvage in cancer surgery in general [27]. The authors found only observational studies, most of which compare patients that received unfiltered or filtered salvaged blood with those who did not undergo cell salvage. Despite the fact that patients who received autologous salvaged blood with or without leucodepletion filters showed a reduced risk of cancer recurrence compared to control groups, the observational nature of these studies forced the authors to underline the low strength of evidence. They conclude by underlining the urgency of multi-center randomized controlled trials comparing mortality and cancer recurrence rates of cell salvage with or without filtration versus allogeneic blood transfusion.

## 5. Conclusions

According to national and international medical guidelines (i.e., the SIMTI, EDQM and NICE guidelines) [1,16,38], cell salvage can be applied to cancer surgery, taking into account patient characteristics and local protocols. In many countries, leucodepletion of salvaged blood is not required and autologous blood could theoretically be re-infused immediately after washing. Nevertheless, several guidelines recommend the use of leucodepletion filters, as for instance in the European Union and the UK [15,31,38], while the Italian guidelines are among the most precautionary, recommending also the subsequent irradiation [16]. In this context, our in vitro investigation represents a proof-of-concept study confirming that cell salvage reduces the number of cancer cells as well as affects proliferation capacity. Further clinical studies must be conducted to verify the safety of washed blood in terms of the risk of reinfusion of cancer cells.

## Figures and Tables

**Table 1:** Patient demographic and characteristics of the cell salvage simulation.

Patient Population	
N (males, females)	12 (4, 8)
Age (mean ± SD)	70.4 ± 10.1
Primary diagnosis	
Colorectal cancer (N)	4
Hepatocarcinoma (N)	8
Presence of diffused metastases (N/total)	5/12
Surgery	
Type of surgery	Partial hepatectomy
Duration (mean ± SD) hours	2.2 ± 0.9
Whole blood recovered (mean ± SD) mL	1021 ± 519
Washed RBCs (mean ± SD) mL	115 ± 80
Filtered RBCs (mean ± SD) mL	70 ± 50

**Table 2:** CD45^−^/EpCAM^+^ cell counts measured in cancer patients. Data were collected at least in duplicate and are shown as mean cells/μL ± SD.

Patient ID	Negative Control		Reservoir		Washed		LD	
	Mean	SD	Mean	SD	Mean	SD	Mean	SD
1	n.d.	n.d.	10.51	2.19	0.53	0.2	0.28	0.04
2	0.91	0.1	26.35	0.35	4.87	0.01	1	0.01
3	2.47	0.13	25.76	4.33	12.49	1.22	0.91	0.11
4	3.62	0.12	4.4	0.11	2.12	0.46	1.41	0.03
5	n.d.	n.d.	7.27	1.1	16.35	4.67	n.d.	n.d.
6	0.33	0.11	3.29	0.71	1.79	0.16	n.d.	n.d.
7	21.3	2.11	63.75	11.58	32.4	10.95	1.58	0.58
8	9.16	0.71	12.25	0.1	7.02	0.34	3.68	0.02

n.d. = not defined.

## Data Availability

The data presented in this study are all available in the article and in the related supplementary material.

## Supplementary Materials

The following supporting information can be downloaded at: https://www.mdpi.com/article/10.3390/jcm12124088/s1, Figure S1: PRISMA flow diagram of the literature revision; Table S1: Clinical and in vitro/ex vivo studies on cell salvage plus filtration and/or irradiation in cancer surgery published since 2013 [14,17,18,20,30,32,33,34,35,36,37,39,40,41,42,43,44,45,46,47]; Table S2: Reviews, systematic reviews and meta-analyses on cell salvage plus filtration and/or irradiation in cancer surgery published since 2013 [27,48,49,50,51]; Table S3: Blood count parameters collected from in vitro cell salvage steps; Table S4. EpCAM-positive cells counts assessed by means of flow cytometry.
