# Supplementary material for: Reduction of EpCAM-Positive Cells from a Cell Salvage Product Is Achieved by Leucocyte Depletion Filters Alone
Source: J Clin Med. 2023 Jun 16;12(12):4088. doi: 10.3390/jcm12124088 (PMC10299373; doi:10.3390/jcm12124088)
Supplement: Supplementary file 1 [file jcm-12-04088-s001.zip › jcm-2325136-supplementary.pdf]

Supplementary Information

Reduction of EpCAM-Positive Cells from a Cell Salvage Product Is Achieved by Leucocyte Depletion Filters Alone

Lucia Merolle [1], Davide Schiroli [1,*], Daniela Farioli [1], Agnese Razzoli [1,2], Gaia Gavioli [1,2], Mauro Iori [3], Vando Piccagli [3], Daniele Lambertini [3], Maria Chiara Bassi [4], Roberto Baricchi [1] and Chiara Marraccini [1]

[1] Transfusion Medicine Unit, Azienda USL-IRCCS di Reggio Emilia, 42123 Reggio Emilia, Italy
[2] Clinical and Experimental Medicine PhD Program, University of Modena and Reggio Emilia, 41125 Modena, Italy
[3] Medical Physics Unit, Azienda USL—IRCCS di Reggio Emilia, 42123 Reggio Emilia, Italy
[4] Medical Library, Azienda USL—IRCCS di Reggio Emilia AUSL-IRCCS di Reggio Emilia, 42123 Reggio Emilia, Italy
* Correspondence: davide.schiroli@ausl.re.it; Tel.: +39-0522-295057

Supplementary Materials and Methods

Literature Review

A bibliographic search was carried out on the MEDLINE, Embase and Scopus databases from 2012 to 2022. A search string containing the following terms and limits was generated: ((("Salvage Therapy"[MeSH] OR "Operative Blood Salvage"[Mesh] OR cell salvage[Title/Abstract] OR blood salvage[Title/Abstract]) AND (("Neoplasms"[Mesh] OR cancer[Title/Abstract] OR tumor[Title/Abstract] OR neoplasm[Title/Abstract] OR oncol*[Title/Abstract]))) AND ((filtration[title/abstract] OR leukocyte depletion[title/abstract] OR LDF[title/abstract]))) OR ((("Salvage Therapy"[MeSH] OR "Operative Blood Salvage"[Mesh] OR cell salvage[Title/Abstract] OR blood salvage[Title/Abstract]) AND (("Neoplasms"[Mesh] OR cancer[Title/Abstract] OR tumor[Title/Abstract] OR neoplasm[Title/Abstract] OR oncol*[Title/Abstract]))) AND (irradiation[title/abstract])). A consultation of the Italian regulations, SIMTI national guidelines and the European Directorate for the Quality of Medicines & HealthCare (EDQM) was also carried out.

Results of the literature review

The bibliographic search was focused on the scientific literature published in the last 10 years (since 2013) and concerning cell savage studies in cancer that included a filtration and/or irradiation step. In our search we excluded all the literature that did not assess at least one of these steps. The string reported the presence of 403 articles pertaining to our research question, with 333 articles from PubMed, 59 from Embase, 62 from Cochrane and 46 from Scopus (see PRISMA flow diagram in

Figure SI of the Supplementary Information). Eight reviews were identified and discussed separately. After duplicates removal, sorting for relevance and abstracts/posters exclusion, the full-text articles published in the last 10 years and included in our review were only 20: of these, 7 were clinical (mainly retrospective) and 13 were in vitro/ex vivo studies.

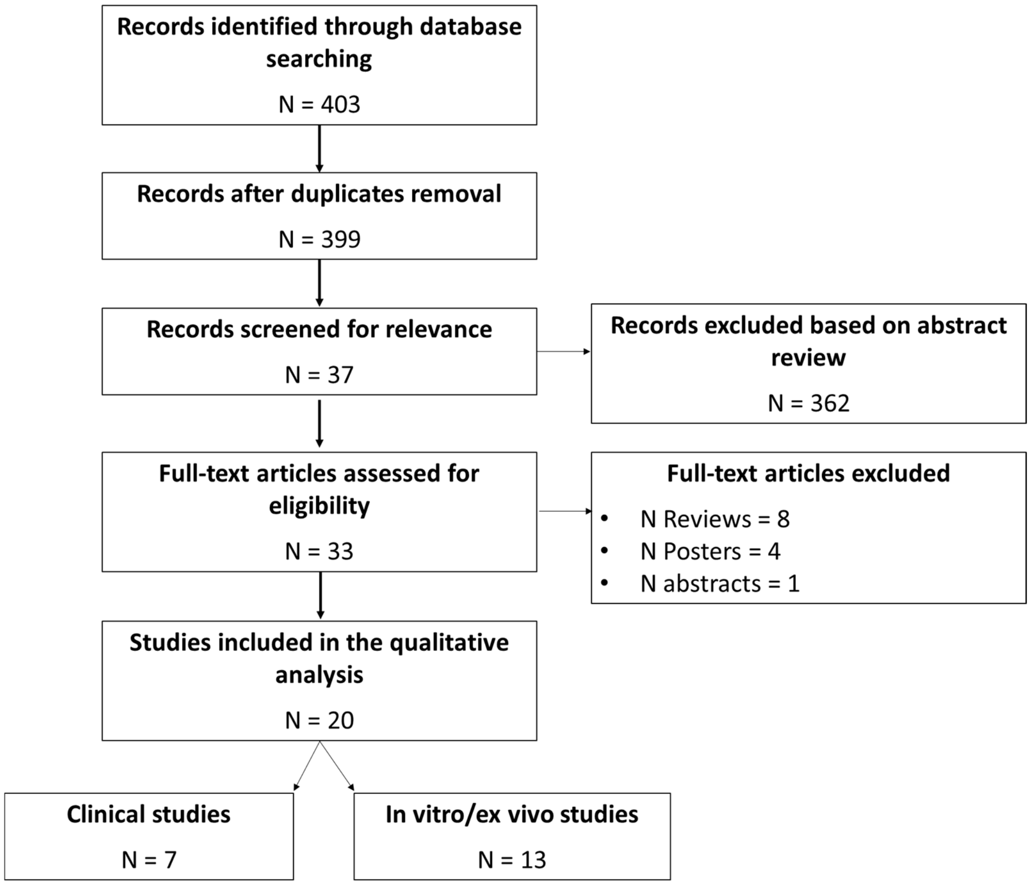

Figure S1. PRISMA flow diagram of the literature revision. Records were identified according to the string detailed in Materials and Methods section.

SUPPLEMENTARY TABLES

Table S1. Clinical and in vitro/ex vivo studies on cell salvage plus filtration and/or irradiation in cancer surgery published since 2013:

Author	Year	Journal	DOI	Study type	Diagnosis	N subjects	Control group	Treatment	N control	N treated
Gakhar H. et al. [1]	2013	Asian Spine Journal	10.4184/asj.2013.7.3.167	clinical	metastatic spine tumor	10	none	CS+LD	none	10
Kim J.M. et al. [2]	2013	Transpl Int	10.1111/tri.12001	clinical	hepatocarcinoma	109	AT	CS+LD	109	121
Han S. et al. [3]	2016	Ann Surg	10.1097/SLA.0000000000001486	clinical	hepatocarcinoma	319	no CS	CS+LD	97	222
Elmalky M. et al. [4]	2017	Spine J	10.1016/j.spinee.2017.03.004	clinical	metastatic spine tumor	176	no CS	CS+LD	113	63
Myrga J.M. et al. [5]	2020	Urology	10.1016/j.urology.2019.08.056	clinical	prostate cancer	157	no CS	CS+LD	70	87
Rajasekaran R.B. et al. [6]	2021	J Bone Oncol	10.1016/j.jbo.2021.100390	clinical	musculoskeletal sarcoma	109	none	CS+LD	none	102
Weller A. et al. [7]	2021	Transplant Proc	10.1016/j.transproceed.2021.03.025	clinical	hepatocarcinoma	51	no CS	CS or CS+IRR	12	11 + 28
Kumar N. et al. [8]	2014	Ann Surg Oncol	10.1245/s10434-014-3569-x	ex vivo	metastatic spine tumour	24	CS	CS+LD	24	24
Kumar N. et al. [9]	2014	Ann Surg Oncol	10.1245/s10434-014-3950-9	ex vivo	metastatic spine tumour	11	CS	CS+LD	11	11
Kumar N. et al. [10]	2016	Eur Spine J	10.1007/s00586-015-4112-x	ex vivo	metastatic spine tumour	50	CS	CS+LD	50	50
Kumar N. et al. [11]	2016	Eur Spine J	10.1007/s00586-016-4478-4	ex vivo	metastatic spine tumour	12	CS	CS+LD	12	12
Tan J.K.H. et al. [12]	2021	HPB (Oxford)	10.1016/j.hpb.2021.04.006	ex vivo	hepatocarcinoma	15	CS	CS+LD	15	15
Winter A. et al. [13]	2021	BMC Anesthesiol	10.1186/s12871-021-01479-3	ex vivo	cancer	16	LD	Catuvab ™	16	16
Zhang X. et al. [14]	2021	BMC Urol	10.1186/s12894-021-00803-w	ex vivo	renal cell carcinoma	5	CS	CS+LD	5	5
Zong Y.N. et al. [15]	2022	BMC Anesthesiol	10.1186/s12871-022-01743-0	ex vivo	metastatic spine tumour	20	CS+LR	CS+LD	12	8

Gong M. et al. [16]	2015	PLoS One	10.1371/journal.pone.0127181	in vitro	cancer cell lines	14	no IRR	IRR	14	14
Mei K. et al. [17]	2015	PLoS One	10.1371/journal.pone.0130864	in vitro	HepG2 and primary cells	30	CS+LR	CS+LD	30	30
Marraccini C. et al. [18]	2017	Vox Sanguinis	10.1111/vox.12565	in vitro	HCT116 cell line	10	CS	CS+LD	10	10
Zhang F.J. et al. [19]	2017	Sci Rep	10.1038/s41598-017-08405-z	in vitro	HCC cell lines	14	IRR 30 Gy	IRR 50 Gy	14	14
Yu Y. et al. [20]	2019	Transfusion	10.1111/trf.15499	in vitro	HCT116 cell line	10	LD	IRR+Riboflavin	10	10

** CS = cell salvage; AT = allogenic transfusion; LR = leucoreduction; LD = leucodepletion; IRR = irradiation; CS+LR = cell salvage plus leucoreduction; CS+LD = cell salvage plus leucodepletion; CS+IRR = cell salvage plus irradiation*

Table S2. Reviews, systematic reviews and meta-analyses on cell salvage plus filtration and/or irradiation in cancer surgery published since 2013. Reviews published in languages other than English were excluded:

Author	Year	Journal	DOI	Review type	Type of surgery	Title
Zhai B. et al. [21]	2013	World J Gastroenterol	10.3748/wjg.v19.i22.3371	Review	liver transplantation	Controversy over the use of intraoperative blood salvage autotransfusion during liver transplantation for hepatocellular carcinoma patients
Kumar N. et al. [22]	2014	Lancet Oncol	10.1016/S1470-2045(13)70245-6	Systematic review	spine surgery	Use of intraoperative cell-salvage for autologous blood transfusions in metastatic spine tumour surgery: a systematic review
Ferroni M.C. et al. [23]	2017	Rev Urol	10.3909/riu0721	Review	urologic surgery	The use of intraoperative cell salvage in urologic oncology
Kumar N. et al. [24]	2018	Neurospine	10.14245/ns.1836140.070	Review	spine surgery	Current Status of the Use of Salvaged Blood in Metastatic Spine Tumour Surgery
Frietsch T. et al. [25]	2022	Transfus Med Hemother	10.1159/000524538	Systematic review & meta-analysis	cancer surgery in general	Safety of Intraoperative Cell Salvage in Cancer Surgery: An Updated Meta-Analysis of the Current Literature

Table S3. Blood count parameters collected from in vitro cell salvage steps. Data are expressed as mean ± SD:

	HGB (g/dL)		HCT (%)		WBC (10^3/uL)		PLT (10^3/uL)	
	**mean**	**SD**	**mean**	**SD**	**mean**	**SD**	**mean**	**SD**
**Whole Blood**	15.2	3.0	44.8	44.8	2.6	0.5	129.8	40.4
**Reservoir**	14.4	2.9	42.6	8.3	2.2	0.4	114.9	36.6
**Washed**	22.0	3.4	62.2	8.0	2.9	1.1	27.3	8.9
**LD**	19.9	1.3	59.6	3.4	0.0	0.0	0.9	0.9
**LD+IRR**	20.0	1.3	60.0	3.0	0.0	0.0	1.3	1.0

Table S4. EpCAM-positive cells counts assessed by means of flow cytometry. Data are expressed as mean cells/ L ± SD:

	CaCo-2		HCT-116	
	**mean**	**SD**	**mean**	**SD**
**Inoculation**	44.3	4.1	45.6	6.1
**Washed**	2.7	2.1	2.0	1.1
**LD**	0.3	0.2	0.3	0.3
**LD+IRR**	0.0	0.0	0.1	0.1

References

1. Gakhar, H.; Bagouri, M.; Bommireddy, R.; Klezl, Z. Role of intraoperative red cell salvage and autologus transfusion in metastatic spine surgery: a pilot study and review of literature. *Asian Spine J.* **2013**, *7*, 167–172, doi: 10.4184/asj.2013.7.3.167.

2. Kim, J.M.; Kim, G.S.; Joh, J.W.; Suh, K.S.; Park, J.B.; Ko, J.S.; Kwon, C.H.; Yi, N.J.; Gwak, M.S.; Lee, K.W.; et al. Long-term results for living donor liver transplantecipients with hepatocellular carcinoma using intraoperative blood salvage with leukocyte depletion filter. *Transpl. Int*. **2013**, *26*, 84–89.

3. Han, S.; Kim, G.; Ko, J.S.; Sinn, D.H.; Yang, J.D.; Joh, J.W.; Lee, S.K.; Gwak, M.S. Safety of the Use of Blood Salvage and Autotransfusion During Liver Transplantation for Hepatocellular Carcinoma. *Ann Surg*. **2016**, *264*, 339-343, doi: 10.1097/SLA.0000000000001486.

4. Elmalky, M.; Yasin, N.; Rodrigues-Pinto, R.; Stephenson, J.; Carroll, C.; Smurthwaite, G. et al. The safety, efficacy, and cost-effectiveness of intraoperative cell salvage in metastatic spine tumor surgery. *Spine J*. **2017**, *17*, 977–982.

5. Myrga, J.M.; Ayyash, O.M.; Bandari, J.; Fam, M.M.; Macleod, L.C.; Jacobs, B.L.; Davies, B.J. The Safety and Short-term Outcomes of Leukocyte Depleted Autologous Transfusions During Radical Cystectomy. *Urology* **2020**, *135*, 106–110.

6. Rajasekaran, R.B.; Palmer, A.J.R.; Whitwell, D.; Cosker, T.D.A.; Pigott, D.; Zsolt, O.; Booth, R.; Gibbons, M.R.J.P.; Carr, A. The role of intraoperative cell salvage for musculoskeletal sarcoma surgery. *J. Bone Oncol*. **2021**, *30*, 100390, doi: 10.1016/j.jbo.2021.100390.

7. Weller, A.; Seyfried, T.; Ahrens, N.; Baier-Kleinhenz, L.; Schlitt, H.J.; Peschel, G.; Graf, B.M.; Sinner, B. Cell Salvage Cell Salvage During Liver Transplantation for Hepatocellular Carcinoma: A Retrospective Analysis of Tumor Recurrence Following Irradiation of the Salvaged Blood. *Transpl. Transplant. Proc.* **2021**, *53*, 1639–1644.

8. Kumar, N.; Ahmed, Q.; Lee, V.K.M.; Chen, Y.; Zaw, A.S.; Goy, R.; Agrawal, R.V.; Dhewar, A.N.; Wong, H.K. Can There be a Place for Intraoperative Salvaged Blood in Spine Tumor Surgery? *Ann. Surg. Oncol.* **2014**, *21*, 2436–2443.

9. Kumar, N.; Lam, R.; Zaw, A.S.; Malhotra, R.; Tan, J.; Tan, G.; Setiobudi, T. Flow cytometric evaluation of the safety of in-traoperative salvaged blood filtered with leucocyte depletion filter in spine tumour surgery. *Ann. Surg. Oncol*. **2014**, *21*, 4330–4335.

10. Kumar, N.; Ahmed, Q.; Lee, V.K.; Zaw, A.S.; Goy, R.; Wong, H.K. Are we ready for the use of intraoperative salvaged blood in metastatic spine tumour surgery? *Eur. Spine J.* **2016**, *25*, 3997–4007.

11. Kumar, N.; Zaw, A.S.; Khoo, B.L.; Nandi, S.; Lai, Z.; Singh, G.; Lim, C.T.; Thiery, J.P. Intraoperative cell salvage in metastatic spine tumour surgery reduces potential for reinfusion of viable cancer cells. *Eur. Spine J*. **2016**, *25*, 4008–4015.

12. Tan, J.K.H.; Menon, N.V.; Tan, P.S.; Pan, T.L.T.; Bonney, G.K.; Shridhar, I.G.; Madhavan, K.; Lim, C.T.; Kow, A.W.C. Presence of tumor cells in intra-operative blood salvage autotransfusion samples from hepatocellular carcinoma liver transplantation: Analysis using highly sensitive microfluidics technology. *HPB* **2021**, *23*, 1700–1707.

13. Winter, A.; Zacharowski, K.; Meybohm, P.; Schnitzbauer, A.; Ruf, P.; Kellermann, C.; Lindhofer, H. Removal of EpCAM-positive tumor cells from blood collected during major oncological surgery using the Catuvab device- a pilot study. *BMC Anesthesiol*. **2021**, *21*, 261.

14. Zhang, X.; Guo, X.; Zong, Y.; Xu, C.; Wang, J.; Zhang, B.; Liu, C.; Gong, Y.; Xue, L.; Ma, L.; et al. CTCs detection from in-traoperative salvaged blood in RCC-IVC thrombus patients by negative enrichment and iFISH identification: A preliminary study. *BMC Urol.* **2021**, *21*, 89.

15. Zong, Y.-N.; Xu, C.-Y.; Gong, Y.-Q.; Zhang, X.-Q.; Zeng, H.; Liu, C.; Bin Zhang, B.; Xue, L.-X.; Guo, X.-Y.; Wei, F.; et al. Effectiveness of intraoperative cell salvage combined with a modified leucocyte depletion filter in metastatic spine tumour surgery. *BMC Anesth. Anesthesiol*. **2022**, *22*, 217.

16. Gong, M.; Yang, J.T.; Liu, Y.Q.; Tang, L.H.; Wang, Y.; Wang, L.J.; Zhang, F.-J.; Yan, M. Irradiation Can Selectively Kill Tumor Cells while Preserving Erythrocyte Viability in a Co-Culture System. *PLoS ONE* **2015**, *10*, e0127181.

17. Mei, K.; Du, L.; Yan, M.; Zhang, Z.; Zhang, F.; Gong, L.; Sun, K.; Zhang, J.; Tang, Y.; Jiang, C.; et al. Modified Leukocyte Filter Removes Tumor Cells from the Salvaged Blood. *PLoS ONE* **2015**, *10*, e0130864.

18. Marraccini, C.; Merolle, L.; Berni, P.; Boito, K.; Tamagnini, I.; Kuhn, E.; Ragazzi, M.; Baricchi, R.; Pertinhez, T.A. Safety of leucodepleted salvaged blood in oncological surgery: An in vitro model. *Vox Sang*. **2017**, *112*, 803–805.

19. Zhang, F.J.; Yang, J.T.; Tang, L.H.; Wang, W.N.; Sun, K.; Ming, Y.; Muhammad, K.G.; Zheng, Y.F.; Yan, M. Effect of X-ray irradiation on hepatocarcinoma cells and erythrocytes in salvaged blood. *Sci Rep*. **2017**, *7*, 7995.

20. Yu, Y.; Yang, L.; He, C.; Tai, S.; Ma, C.; Yang, T.; Wang, D. Evaluation of riboflavin photochemical treatment for inactivation of HCT116 tumor cells mixed in simulative intraoperative salvage blood. *Transfusion* **2019**, *59*, 3205–3213.

21. Zhai, B.; Sun, X.Y. Controversy over the use of intraoperative blood salvage autotransfusion during liver transplantation for hepatocellular carcinoma patients. *World J. Gastroenterol*. **2013**, *19*, 3371–3374.

22. Kumar, N.; Chen, Y.; Zaw, A.S.; Nayak, D.; Ahmed, Q.; Soong, R.; Wong, H.K. Use of intraoperative cell-salvage for autologous blood transfusions in metastatic spine tumour surgery: a systematic review. *Lancet Oncol*. **2014,** *15*, e33–41.

23. Ferroni, M.C.; Correa, A.F.; Lyon, T.D.; Davies, B.J.; Ost, M.C. The use of intraoperative cell salvage in urologic oncology. *Rev. Urol*. **2017**, *19*, 89–96.

24. Kumar, N.; Ravikumar, N.; Tan, J.Y.H.; Akbary, K.; Patel, R.S.; Kannan, R. Current Status of the Use of Salvaged Blood in Metastatic Spine Tumour Surgery. *Neurospine*. **2018**, *15*, 206–215.

25. Frietsch, T.; Steinbicker, A.U.; Horn, A.; Metz, M.; Dietrich, G.; Weigand, M.A.; Waters, J.H.; Fischer, D. Safety of Intraoperative Cell Salvage in Cancer Surgery: An Updated Meta-Analysis of the Current Literature. *Transfus. Med. Hemother* **2022**, *49*, 143–157.
